# Impact of High-Intensity Interval Training on Aerobic Capacity, Muscle Strength, and Agility Among Football Athletes: A Systematic Review and Meta-Analysis

**DOI:** 10.3390/sports14090398

**Published:** 2026-09-09

**Authors:** Himani Sanjay Vartak, Jorge Costa, Sampath Kumar Amaravadi

**Affiliations:** School of Sport, Exercise and Rehabilitation Sciences, University of Birmingham, Birmingham B15 2TT, UK; himanivartak66@gmail.com (H.S.V.); j.costa@bham.ac.uk (J.C.)

**Keywords:** high-intensity interval training, football, aerobic fitness, VO_2_max, muscle strength, agility

## Abstract

High-intensity interval training (HIIT) is increasingly incorporated into football conditioning programmes to enhance aerobic fitness, muscular strength, and agility. This systematic review and meta-analysis evaluated the effects of HIIT on these performance outcomes in football players and explored the availability of evidence relating to injury outcomes. Six electronic information sources were searched for randomised and non-randomised controlled trials published between January 2010 and December 2024. Methodological quality was assessed using the Modified Downs and Black checklist, and certainty of evidence was evaluated using GRADE. Ten studies including 329 football players were included. Aerobic-fitness outcomes were synthesised narratively because heterogeneity in outcome measurement and reporting precluded reliable quantitative pooling. Individual studies demonstrated favourable changes, although superiority over comparator training was not consistently established. Muscle-strength outcomes were also synthesised narratively because sufficiently comparable independent-study data were unavailable. Two RCTs contributed to the agility meta-analysis; following harmonisation of the direction of time-based outcomes, no significant advantage of HIIT over comparator training was observed (SMD = −0.29, 95% CI −0.83 to 0.25; *p* = 0.290). Certainty of evidence was low across performance outcomes. No included study directly assessed injury incidence. Overall, HIIT may contribute to improvements in football-related physical performance; however, the limited, heterogeneous, and non-professional evidence base warrants cautious interpretation and further adequately powered trials.

## 1. Introduction

Football is a physically demanding, intermittent sport that requires high levels of aerobic and anaerobic fitness, muscular strength, agility and tactical awareness. During a standard 90 min match, players typically cover between 8 and 12 km while performing repeated bouts of high-intensity activity interspersed with periods of lower-intensity activity and recovery [1]. The ability to sustain and repeatedly perform these efforts is influenced by aerobic fitness, with positional differences evident. For example, midfielders cover greater total distances and expend more energy than defenders or forwards [2,3]. High-intensity running is also an important component of match performance, with wide midfielders reported to cover more than 1000 m at high intensity, compared with approximately 850 m among centre forwards [3].

Alongside these substantial physical demands, football is associated with a considerable burden of injury [4]. Muscle strains, ligament injuries and joint-related conditions can result in absence from training and competition, disrupt performance and, in some cases, influence longer-term participation [5,6]. Physical characteristics such as aerobic fitness, muscular strength and movement control may be relevant to some mechanisms associated with injury risk. However, improvements in these characteristics should not be assumed to translate directly into reductions in injury incidence. Demonstrating an injury-prevention effect requires studies that directly assess outcomes such as injury incidence, injury burden, time-loss injury or reinjury.

High-intensity interval training (HIIT) has gained increasing attention as a time-efficient conditioning strategy characterised by repeated bouts of high-intensity exercise interspersed with periods of active or passive recovery [7]. This intermittent structure has clear relevance to football, where players repeatedly accelerate, decelerate, sprint and change direction throughout match play. HIIT can be delivered through running-based intervals, sprint protocols or football-specific activities, and previous studies have investigated its effects on aerobic fitness, maximal oxygen uptake (VO_2_max), muscular performance and agility [8,9,10].

The physiological mechanisms underpinning these adaptations include increased cardiac output, greater capillary density, improved mitochondrial biogenesis and enhanced recruitment of type II fast-twitch muscle fibres [11,12,13,14]. Neuromuscular adaptations may also improve coordination, proprioception and reactive agility, which are essential for effective performance and injury resilience in football. Consistent application of HIIT, typically two to three sessions per week for at least six weeks, can lead to significant improvements in endurance and strength. However, evidence remains limited regarding its long-term benefits and specific contribution to injury prevention among football players [1].

Despite the widespread use of HIIT in football, evidence regarding its effects on key football-related performance outcomes remains heterogeneous. Previous systematic review evidence has demonstrated variation in HIIT protocols, participant characteristics, comparator conditions and outcome measures, with the magnitude of effects differing across aerobic and other physical-performance outcomes [15]. Furthermore, although improvements in aerobic fitness, muscular strength and neuromuscular performance may theoretically influence factors associated with injury risk, direct evidence that HIIT itself reduces injury incidence in football remains limited. Current evidence for reducing football injuries has predominantly been derived from dedicated multicomponent neuromuscular injury-prevention programmes, such as the FIFA 11+, rather than from HIIT interventions specifically [16].

Accordingly, the primary objective of this systematic review and meta-analysis was to evaluate the effects of HIIT on aerobic fitness, muscular strength and agility among football players across different competitive levels. A secondary exploratory objective was to determine whether the included literature provided direct evidence regarding injury outcomes and, where direct evidence was unavailable, to identify the limits of any injury-related interpretation.

The review addressed the following questions:

1. What are the effects of HIIT on aerobic fitness, muscular strength and agility in football players across different competitive levels?
2. Do the included HIIT studies provide direct evidence regarding injury outcomes in football players, and what injury-related implications, if any, can be considered only exploratory in the absence of direct injury data?

## 2. Materials and Methods

This systematic review and meta-analysis was registered with the Open Science Framework (OSF) Registry (https://doi.org/10.17605/OSF.IO/4JM7Z) [17] and conducted according to the Preferred Reporting Items for Systematic Reviews and Meta-Analyses (PRISMA) 2020 guidelines [18,19] (Appendix A, Table A1). The design of the study was intended to ensure methodological rigour, transparency and reproducibility, allowing for reliable synthesis of the available evidence.

### 2.1. Literature Identification

The literature search covered the period from 1 January 2010 to 31 December 2024. The lower date limit was selected to focus the review on contemporary HIIT interventions using structured exercise-prescription approaches and outcome assessments that are more representative of current football-conditioning practice. This temporal restriction was not intended to imply an absence of the relevant interval-training literature before 2010, and the potential exclusion of earlier evidence was considered when interpreting the findings. The following databases were searched: PubMed/MEDLINE, CINAHL Plus, ProQuest, SPORT Discus, Google Scholar and the Sports Medicine and Education Index, with database-specific adaptations where required. Search terms were organised around three principal concepts: (1) HIIT, (2) football, and (3) eligible performance outcomes. Synonymous terms within each concept were combined using the Boolean operator OR, while the principal concepts were combined using AND. Importantly, aerobic fitness, muscular strength and agility were combined using OR so that studies reporting any one or more of these eligible outcomes could be retrieved. The complete database-specific search strategies, including all search terms, Boolean operators, database-specific adaptations and applied limits, are presented in Appendix A, Table A2. The core search structure was (“high-intensity interval training” OR HIIT OR “high intensity interval training” OR “interval training”) AND (football OR footballer OR soccer OR “soccer player”) AND (“aerobic capacity” OR “aerobic fitness” OR VO_2_max OR “maximal oxygen uptake” OR “muscle strength” OR “muscular strength” OR “isokinetic strength” OR agility OR “change of direction”). Search syntax was adapted as required for each database while retaining these three conceptual domains. Reference lists of included studies were additionally screened to identify potentially eligible studies.

Two independent reviewers (HV and SA) conducted the screening process. This involved initial title screening, followed by abstract and full-text assessment against the eligibility criteria, while discrepancies were resolved through discussion.

#### 2.1.1. Inclusion Criteria and Exclusion Criteria

Studies were eligible for inclusion if they were randomised controlled trials (RCTs) or non-randomised controlled trials (NRCTs) involving male or female football players aged 13–35 years from amateur, semi-professional or professional competitive levels. Both RCTs and NRCTs were included because the available intervention literature across the prespecified football-performance outcomes was limited, and inclusion of controlled non-randomised evidence enabled a broader assessment of relevant HIIT interventions. However, because NRCTs are more susceptible to confounding, selection bias and other design-related limitations than adequately conducted RCTs, study design was explicitly considered during methodological appraisal, evidence synthesis and interpretation. Cochrane similarly notes the greater potential for bias in non-randomised intervention studies and recommends cautious interpretation when they are included.

Eligible interventions involved HIIT lasting between 4 and 12 weeks and reported at least one primary outcome related to aerobic fitness, muscular strength or agility. Aerobic-fitness outcomes included VO_2_max or comparable validated aerobic-performance measures; muscular-strength outcomes included isokinetic strength or one-repetition maximum measures; and agility outcomes included validated change-of-direction or agility tests. Injury outcomes were not required for study inclusion and were considered only as a secondary exploratory outcome.

RCT and NRCT findings were not automatically pooled together. Quantitative synthesis was undertaken only when studies were sufficiently comparable in population, intervention/comparator characteristics and outcome measurement. Where quantitative pooling was inappropriate, findings were synthesised narratively. Greater interpretive weight was given to evidence from randomised trials, while NRCT findings were considered supportive and interpreted considering their methodological limitations and the corresponding GRADE certainty assessment.

#### 2.1.2. Types of Intervention

Eligible interventions were required to last between 4 and 12 weeks. Among the studies included in the review, the observed intervention durations ranged from 4 to 10 weeks, as no eligible study with a duration longer than 10 weeks met the inclusion criteria. Studies that compared HIIT vs. moderate-intensity continuous training or HIIT vs. control groups were included.

#### 2.1.3. Types of Outcome Measures

The primary outcome measures for the systematic review were aerobic capacity (VO2max), muscle strength measured via an isokinetic dynamometer (°/s Nm) and one-repetition maximum weight (in kgs), and agility performance measured over time.

### 2.2. Methodological Quality Assessment

The methodological quality of all included studies was independently assessed by two reviewers using the Modified Downs and Black checklist [20]. This checklist was selected to provide a consistent appraisal framework applicable to both randomised controlled trials (RCTs) and non-randomised controlled trials (NRCTs). The assessment considered reporting quality, external validity, internal validity related to bias, internal validity related to confounding and statistical power. Total scores were categorised according to the predefined modified scoring criteria as excellent (26–28), good (20–25), fair (15–19) or poor (≤14). Disagreements between reviewers were resolved through discussion and consensus.

Both the overall rating and the pattern of limitations across individual methodological domains were considered when interpreting the findings. The overall Downs and Black quality category was not directly converted into a GRADE certainty rating. Instead, relevant methodological limitations identified during critical appraisal were considered when judging study limitations within the GRADE assessment for each outcome.

### 2.3. Data Extraction

Data were extracted independently by two reviewers (HV and SA) using a standardised data-extraction form based on the JBI approach for systematic reviews [21] (Appendix A, Table A3). Extracted information included study design, participant characteristics, competitive level, sample size, intervention and comparator characteristics, HIIT frequency, intensity and duration, outcome assessment methods, and pre- and postintervention data required for qualitative or quantitative synthesis. Outcome data were extracted separately for aerobic fitness, muscular strength and agility. The two reviewers (HV and SA) independently verified the completeness and accuracy of the extracted data, and disagreements were resolved through discussion and consensus with a third reviewer (JC).

### 2.4. Data Analysis and Evidence Synthesis

A narrative synthesis was initially undertaken for each prespecified performance domain, comprising aerobic fitness, muscular strength and agility. Studies were grouped according to outcome, study design and the comparability of participant characteristics, interventions, comparator conditions and outcome measures.

Meta-analysis was undertaken only when at least two independent studies reported sufficiently comparable outcomes and provided adequate statistical information for calculation of an effect estimate. Standardised mean differences (SMDs) with 95% confidence intervals (CIs) were calculated where conceptually similar outcomes were measured using different scales. A random-effects model was applied because clinical and methodological variation was anticipated across football populations, intervention protocols and study designs [22].

Multiple outcome estimates derived from the same participant sample were not considered independent studies for the purposes of meta-analysis. Where a single study reported several related outcome measurements, these estimates were presented individually and interpreted descriptively rather than pooled as independent study-level effects. Accordingly, the four isokinetic muscle-strength estimates reported by Zhang et al. [3] were treated as within-study effect estimates and were not considered an across-study meta-analysis.

Statistical heterogeneity was evaluated using the I^2^ statistic where quantitative synthesis was undertaken. Values above 50% were considered indicative of potentially substantial heterogeneity; however, heterogeneity estimates from analyses involving only two studies were interpreted cautiously because of their limited precision. Prediction intervals were reported where available to illustrate the potential range of effects in future comparable studies, although these were also interpreted cautiously because of the small number of contributing studies.

Evidence from randomised controlled trials (RCTs) and non-randomised controlled trials (NRCTs) was considered in relation to study design and methodological quality. Studies of different designs were not combined solely because they reported a common outcome. Where NRCT evidence was not appropriate for quantitative pooling, it was incorporated into the narrative synthesis and interpreted cautiously in view of the greater susceptibility of non-randomised studies to confounding, selection bias and other methodological limitations. Greater interpretive weight was therefore placed on evidence from methodologically stronger randomised trials.

Formal assessment of publication bias was not undertaken because the number of studies contributing to each meta-analysis was insufficient for reliable evaluation of funnel-plot asymmetry or statistical testing. Funnel plots and Egger’s regression tests were therefore not used. The possibility of publication and selective-reporting bias was instead acknowledged as a limitation of the available evidence. When meta-analysis was not feasible because of insufficient, heterogeneous or non-comparable data, findings were synthesised narratively. Methodological quality was explicitly considered when interpreting the direction, magnitude and consistency of study findings. As this review analysed data from previously published studies, ethical approval was not required.

The certainty of evidence for each principal outcome was evaluated using the GRADE framework [23]. Certainty judgements were made at the outcome level and considered study limitations, inconsistency, indirectness, imprecision and potential reporting bias. Methodological limitations identified using the Modified Downs and Black checklist informed the assessment of study limitations but were not converted directly into GRADE certainty categories. Where quantitative synthesis included only a subset of studies contributing evidence for an outcome, studies contributing to the pooled estimate were clearly distinguished from those contributing only to the narrative synthesis.

Competitive level was also considered as a potential source of clinical heterogeneity. An exploratory subgroup analysis comparing amateur with semi-professional/professional players was considered; however, subgroup meta-analysis was not undertaken because fewer than two independent studies with sufficiently comparable outcome data were available within the relevant competitive-level subgroups. Competitive level was therefore examined descriptively.

## 3. Results

### 3.1. Study Selection and Characteristics

**Figure 1:**
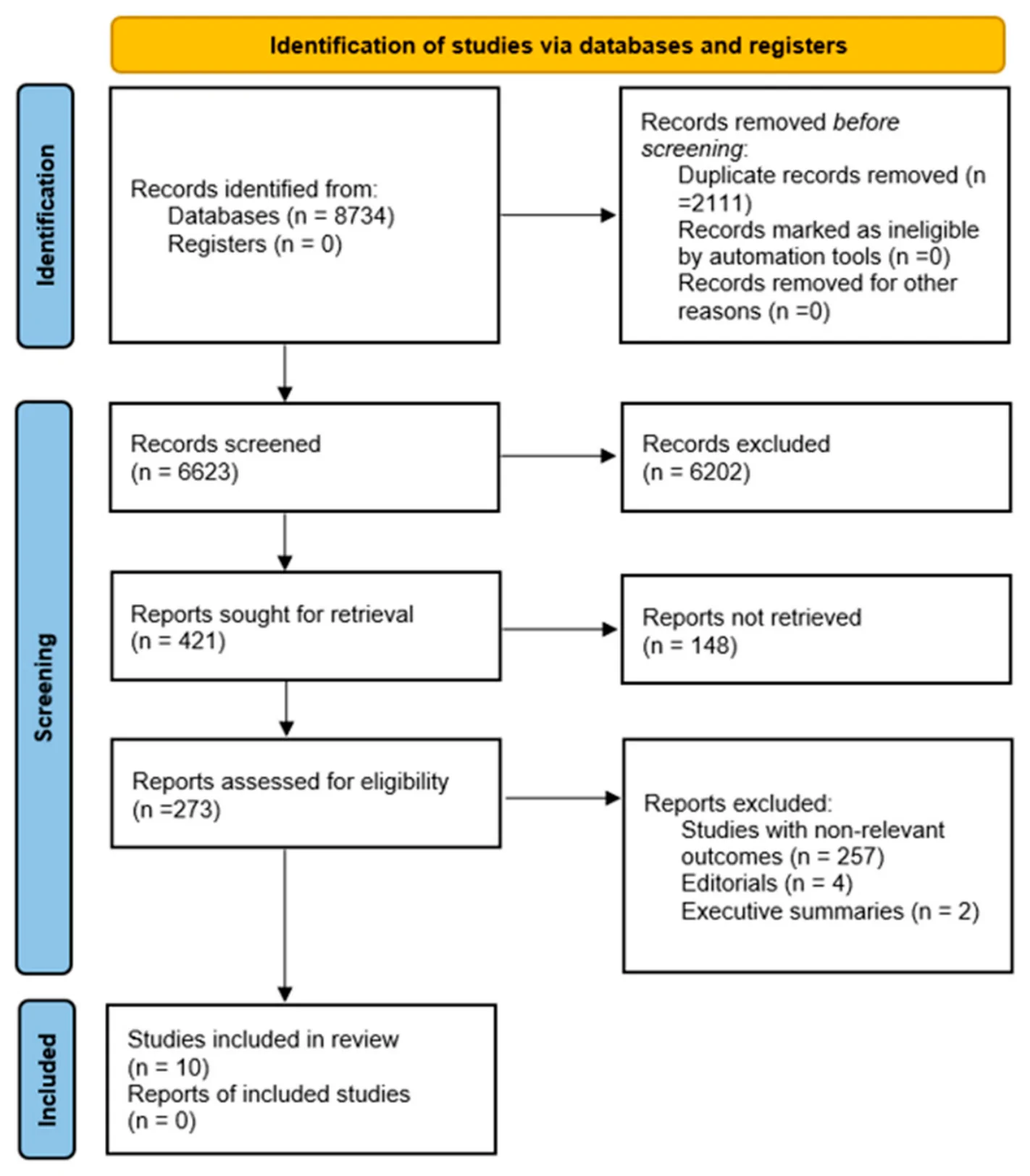
PRISMA 2020 flow diagram illustrating the study identification, screening, eligibility and inclusion process.

The initial database search and reference list screening identified a total of 8734 records. After 2111 duplicates were removed, 6623 studies remained for title and abstract screening. Of these, 6202 were excluded for not meeting the inclusion criteria. A total of 421 full-text articles were subsequently assessed for eligibility, with 411 excluded following detailed review due to irrelevant outcomes, editorial or commentary formats, or insufficient methodological detail. Ultimately, ten studies met the inclusion criteria and were included in the final analysis (Figure 1).

Each included study was reviewed for descriptive variables such as participants’ mean age, competitive level, sample size, study design, research setting, type of control group (standard training), exercise modality and intensity for both the control and experimental conditions, duration of the intervention, and outcome measures. Meta-analyses were performed via MetaAnalysisOnline.com software [22].

In total, the included studies included 329 participants, all of whom were football players ranging from amateur to semi-professional and professional levels [1,2,3,8,14,24,25,26,27,28]. The ten included studies involved 329 football players across different competitive levels. Five studies included amateur or youth-amateur players [1,3,14,25,28], three included semi-professional players [2,26,27] and two included professional players [8,24]. Thus, half of the available evidence was derived from amateur-level populations, whereas professional players were represented in only two studies. Intervention durations among the included studies ranged from four to ten weeks. The predefined outcome measures across studies are summarised in Table 1, and the pre- and postintervention data for aerobic capacity, muscle strength and agility performance are presented in Table 2a–c.

#### 3.1.1. Qualitative Analysis of Pre- and Postintervention Differences

##### Aerobic Capacity: Descriptive Pre- and Post-Intervention Findings

In Table 2a, high-intensity interval training (HIIT) interventions demonstrated variable effects on aerobic capacity, with both modest and substantial gains depending on the training protocol and duration. Thomakos et al. [14] reported improvements in Yo-Yo IR1 performance following both HIIT formats. Distance covered increased from 2083 ± 411 to 2434 ± 345 m following the 10 s/10 s protocol and from 2225 ± 262 to 2418 ± 275 m following the 15 s/15 s protocol. Corresponding estimated VO_2_max values increased from 53.9 ± 3.5 to 56.8 ± 2.9 mL·kg^−1^·min^−1^ and from 55.1 ± 2.2 to 56.7 ± 2.3 mL·kg^−1^·min^−1^, respectively [14]. In contrast, Arazi et al. [2] reported improvements among semi-professional female footballers, with VO_2_max increasing by +6.20 mL·kg^−1^·min^−1^ after heart rate-based HIIT and by +4.40 mL·kg^−1^·min^−1^ after speed-based HIIT, indicating that both prescription methods were effective in enhancing aerobic performance. Similarly, Hamdani et al. [8] reported that national-level players improved VO_2_max by +3.00 (heart rate-based) and +8.70 (speed-based), demonstrating greater responsiveness to sprint-oriented protocols even among elite populations.

Slettalokken et al. [26] reported minor gains when HIIT was performed at low frequency during the off-season, with VO_2_max increasing by +1.77 and +0.60 under HIT 0.5 and HIT 1 conditions, respectively. These results suggest that under reduced training loads, HIIT may help maintain rather than markedly improve aerobic capacity. Conversely, Howard et al. [25] reported small declines in VO_2_max (–1.30) and shuttle run performance (–2.20) following an in-season intervention, possibly influenced by concurrent training and fatigue. However, shuttle run values in a subsequent HIIT protocol showed a small reduction in HIT 0.5 (–2.90) but substantial increases in HIT 1 (+432.0) and a second HIIT set (+326.0), indicating that both training volume and intensity significantly influenced aerobic performance outcomes.

##### Muscle Strength: Descriptive Pre- and Post-Intervention Findings

As shown in Table 2b, HIIT, when combined with resistance training, consistently increased both lower- and upper-body strength. Zhang et al. [3] reported improvements in body-mass-normalised isokinetic knee performance following the four-week intervention. At 60°/s, which represented the strength-oriented assessment, the HIIT group demonstrated a 10.2% improvement compared with 3.3% in the MICT group. At 180°/s, which represented the power-oriented assessment, improvements of 7.6% and 5.5% were reported in the HIIT and MICT groups, respectively. Yan et al. [1] similarly reported favourable changes in normalised isokinetic knee performance following both low- and high-frequency HIIT protocols. Because the original studies used differing normalisation procedures and unit conventions, these outcomes were interpreted descriptively rather than compared quantitatively. In professional players, Wong et al. [24] reported substantial increases in maximal strength, with the back half squat improving by +25.00 kg and the bench press by +5.10 kg, demonstrating the synergistic benefits of integrating structured resistance training within HIIT programmes.

##### Agility: Descriptive Pre- and Post-Intervention Findings

In Table 2c, high-intensity interval training (HIIT) also had a positive effect on agility performance, as reflected by faster completion times and improved movement responsiveness. Gokkurt et al. [27] reported a reduction of –0.04 s in Pro Agility Test times, representing a small yet meaningful improvement in short-directional speed. Wright et al. [28] reported the greatest improvement, with a –0.55 s reduction in the modified test completion time, indicating enhanced neuromuscular coordination and rapid change-of-direction ability. In contrast, Howard et al. [25] reported a negligible change of +0.03 s in the Illinois Agility Test, suggesting that, in certain contexts, agility development may require more targeted change-of-direction training components to elicit significant improvements.

### 3.2. Methodological Quality of Included Studies

Methodological quality was evaluated for all ten included studies using the Modified Downs and Black checklist (Table 3). Based on the predefined total-score categories, two studies were classified as excellent quality, six as good quality, one as fair quality and one as poor quality.

Arazi et al. [2] and Thomakos et al. [14] were rated as excellent quality. Yan et al. [1], Zhang et al. [3], Howard et al. [25], Slettalokken et al. [26], Gokkurt et al. [27] and Wright et al. [28] were classified as good quality. Wong et al. [24] was rated as fair quality, whereas Hamdani et al. [8] was classified as poor quality.

Across the included evidence, reporting quality was generally stronger than domains relating to internal validity, confounding and statistical power. Methodological concerns were more prominent in the non-randomised studies, particularly where allocation procedures, control of potential confounding variables, blinding and reporting of methodological procedures were limited or unclear. Consequently, studies classified as fair or poor quality were interpreted more cautiously within the narrative synthesis and were not considered to provide the same level of confidence as studies with stronger methodological characteristics.

Importantly, the Downs and Black categories were used to describe study-level methodological quality rather than to determine certainty of evidence directly. For GRADE, relevant methodological limitations were considered alongside inconsistency, indirectness, imprecision and potential publication bias for each individual outcome.

#### 3.2.1. Effects of HIIT on Aerobic Capacity

Across the five studies that examined the impact of high-intensity interval training (HIIT) on aerobic capacity, the greatest improvement in VO_2_max (17.3%, ES = 0.71) was observed after a six-week speed-based HIIT programme in semi-professional female football players [2]. Howard et al. [25] reported significant improvements in Yo-Yo IR1 distance over the 10-week intervention period in both the HIIT and traditional endurance-training groups. However, no significant between-group difference was identified, suggesting that HIIT produced aerobic-performance improvements comparable to, rather than superior to, traditional conditioning. Similarly, a 10-week sprint-based, in-season HIIT intervention in high school players led to a 15.6% increase in VO_2_max (ES = 0.68) compared with traditional endurance training [25]. Thomakos et al. [14] reported a 13.8% increase in aerobic capacity (ES = 0.64) following short-format 10 s/10 s and 15 s/15 s HIIT protocols during the competitive season among youth players.

Hamdani et al. [8] reported significant improvements in VO_2_max (12.1%) and beep test scores among professional footballers after an eight-week HIIT programme (ES = 0.59) compared with regular training. In contrast, Slettalokken et al. [26] reported only maintenance of VO_2_max (0.4%) following biweekly HIIT during the off-season, with a marginal effect size (ES = 0.15) and a 6% decline in shuttle run performance, indicating potential deconditioning associated with reduced training frequency.

Overall, the individual studies reported variable changes in aerobic capacity following HIIT, with the magnitude and direction of effects differing according to intervention characteristics, comparator conditions and training context. Although several studies reported improvements following HIIT, these findings should not be interpreted as demonstrating consistent superiority over control conditions because only a subset of studies provided sufficiently comparable data for quantitative synthesis. Therefore, the narrative findings suggest a potential beneficial effect of HIIT on aerobic capacity, but the strength of this conclusion remains uncertain.

##### Quantitative Synthesis of Aerobic Fitness

Aerobic fitness outcomes were synthesised narratively because the available studies differed in the reporting and derivation of aerobic-fitness measures, and sufficiently comparable study-level data could not be verified for a reliable quantitative synthesis. Howard et al. [25] reported Yo-Yo Intermittent Recovery Test Level 1 (Yo-Yo IR1) distance rather than directly measured VO_2_max, whereas other studies reported estimated or directly assessed aerobic-capacity outcomes using different procedures. Consequently, no pooled effect estimate was calculated.

##### Reporting Bias and Overall Interpretation of Quantitative Evidence

Formal assessment of publication bias was not undertaken because the number of studies contributing to each quantitative synthesis was too small to permit meaningful evaluation of funnel-plot asymmetry or formal statistical testing. Therefore, the possibility of publication bias cannot be excluded.

The quantitative evidence was limited by the small number of studies with sufficiently comparable and verifiable data. Aerobic-fitness outcomes were therefore synthesised narratively rather than pooled quantitatively. The agility analysis included only two independent studies and should be interpreted cautiously because of the small evidence base. For muscle strength, multiple effect estimates were available from a single study and were therefore treated as within-study estimates rather than as an across-study meta-analysis. Collectively, these limitations reduce confidence in the precision and generalisability of the quantitative findings.

#### 3.2.2. Effects of HIIT on Muscle Strength

Across the three studies that evaluated the effects of high-intensity interval training (HIIT) on muscular strength, the most substantial improvements were observed among professional football players following an eight-week preseason concurrent strength and HIIT programme. In this study, the one-repetition maximum (1RM) half-square increased by 20.3%, and the bench pressure increased by 7.8%, with overall effect sizes ranging from 0.65 to 0.74 [24]. In amateur youth football players, Zhang et al. [3] reported an 8.1% increase in knee extensor strength and a 7.5% increase in flexor strength after a four-week HIIT intervention combined with resistance training, resulting in a moderate effect size of 0.58 compared with the moderate-intensity continuous training (MICT) group. Similarly, Yan et al. [1] reported that a high-frequency HIIT protocol (five sessions per week) produced a 6.3% improvement in knee torque compared with 3.2% in the low-frequency group, corresponding to a small-to-moderate effect size of 0.46.

Three studies evaluated muscular-strength outcomes following HIIT-based interventions [1,3,24]. Zhang et al. [3] assessed isokinetic knee flexor and extensor strength at different angular velocities and reported generally favourable changes following a four-week HIIT programme combined with resistance training. However, because these outcomes were obtained from the same participant sample, they were treated as multiple measurements within a single study rather than as independent effect estimates.

Yan et al. [1] also reported improvements in isokinetic strength measures following low- and high-frequency HIIT protocols, while Wong et al. [24] reported improvements in one-repetition maximum half-squat and bench-press performance following concurrent HIIT and resistance training. Differences in study design, intervention content and strength-assessment methods prevented meaningful quantitative pooling across independent studies. Accordingly, muscle-strength findings were synthesised narratively, and no pooled estimate of the overall effect of HIIT on muscular strength was calculated.

Although the individual studies reported favourable changes in selected strength outcomes, these findings should be interpreted cautiously because all three studies were non-randomised and some interventions included concurrent resistance training. Therefore, the independent contribution of HIIT to the observed strength adaptations cannot be clearly established.

#### 3.2.3. Effects of HIIT on Agility

Three studies reported generally favourable changes in agility performance following HIIT, although the magnitude of improvement varied across studies and only two studies provided sufficiently comparable data for quantitative synthesis. In the first study, high school football players who completed a 10-week sprint-based HIIT programme demonstrated a 3.2% improvement in Illinois Agility Test performance compared with the control group, with a moderate effect size (ES = 0.66) [25]. In the second study, under-19 semi-professional players who participated in an eight-week HIIT intervention demonstrated a small reduction in Pro-Agility Test completion time from 4.73 ± 0.39 s to 4.69 ± 0.39 s, corresponding to an approximately 0.85% within-group improvement, corresponding to a large effect size (ES = 0.88) [27]. Similarly, Wright et al. [28] reported a 2.7% increase in agility performance on the modified to test following an eight-week mixed-methods HIIT programme among young female footballers, with a moderate effect size (ES = 0.57).

Collectively, the individual studies reported variable changes in agility performance following HIIT. Although favourable within-group changes were reported in some studies, between-group differences were not consistently in favour of HIIT. These findings should therefore be interpreted cautiously, particularly because agility was assessed as completion time, for which lower values represent better performance.

#### 3.2.4. Limited Meta-Analysis of Agility

**Figure 2:**
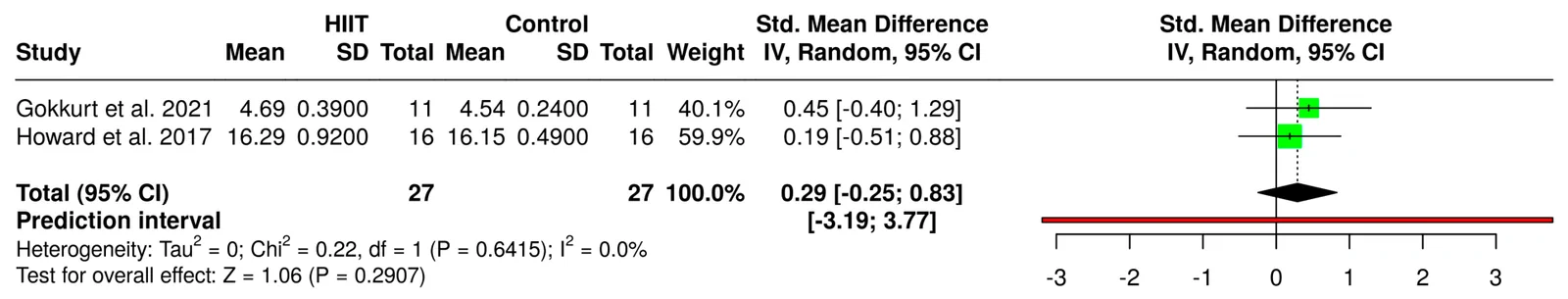
Forest plot of the limited two-study meta-analysis [25,27] examining the effect of HIIT on agility performance in football players. Agility was assessed as completion time, for which lower values indicate better performance. Effect directions were harmonised so that positive values favour HIIT and negative values favour the comparator condition.Individual study markers represent study-level standardised mean differences, with horizontal lines representing 95% confidence intervals. The diamond represents the pooled random-effects estimate and its 95% confidence interval. The vertical reference line at zero represents the null effect.

Two independent RCTs, Howard et al. [25] and Gokkurt et al. [27], provided sufficiently comparable post-intervention agility-time data for quantitative synthesis (Figure 2). Because agility performance was assessed using completion time, for which lower values represent better performance, the direction of effect was harmonised so that positive values represented an effect favouring HIIT and negative values represented an effect favouring the comparator condition.

Following correction of the direction of effect and verification of the study-level data, the pooled standardised mean difference was −0.29 (95% CI −0.83 to 0.25; *p* = 0.290). The point estimate therefore favoured the comparator conditions, although the confidence interval crossed the null and the overall effect was not statistically significant. Statistical heterogeneity was estimated as I^2^ = 0%; however, this estimate should be interpreted cautiously because only two studies contributed to the analysis.

These findings do not provide evidence that HIIT is superior to comparator training for improving agility performance. The wide confidence interval and very small evidence base indicate substantial uncertainty regarding the true effect. Wright et al. [28] provided additional narrative evidence but could not be incorporated into the quantitative synthesis because sufficiently comparable data were unavailable.

### 3.3. Certainty of Evidence (GRADE)

The certainty of evidence was assessed separately for each principal outcome using the GRADE framework (Table 4). The Modified Downs and Black assessment informed consideration of methodological limitations but was not translated directly into GRADE certainty categories.

For aerobic fitness, five studies involving 127 participants contributed to the evidence base, comprising four RCTs [2,14,25,26] and one NRCT [8]. A pooled effect estimate was not calculated because the studies differed in the assessment and reporting of aerobic-fitness outcomes and sufficiently comparable source-verifiable data were unavailable for reliable quantitative synthesis. Individual studies generally reported favourable changes in VO_2_max or field-based aerobic-performance measures following HIIT, although the magnitude and direction of effects varied across intervention protocols and comparator conditions. Importantly, superiority of HIIT over comparator training was not consistently demonstrated. The certainty of evidence was therefore judged to be low because of methodological limitations, small study samples, imprecision and heterogeneity in outcome measurement and intervention characteristics.

Evidence for muscular strength was derived from three NRCTs [1,3,24], involving 143 participants. Because the studies used different strength-assessment methods and intervention designs, and because multiple strength outcomes reported by Zhang et al. [3] originated from a single participant sample, no quantitative pooling was undertaken. Individual studies generally reported favourable changes in selected strength outcomes, but the certainty of evidence was judged to be low because the evidence was entirely non-randomised, study samples were relatively small, outcome measures were heterogeneous, and concurrent resistance-training components limited the extent to which improvements could be attributed specifically to HIIT.

For agility, two RCTs [25,27], involving 54 participants, contributed to the pooled analysis and demonstrated an SMD of 0.77 (95% CI 0.21 to 1.33). The certainty of evidence was judged to be low, with imprecision resulting from the small number of studies and participants representing the principal limitation. Wright et al. [28] additionally contributed supportive narrative evidence but could not be incorporated into the pooled analysis because sufficient comparable data were unavailable.

Certainty of evidence was not graded for injury prevention because none of the included intervention studies directly evaluated injury incidence as an outcome. Accordingly, statements regarding injury prevention should be interpreted as theoretical or indirect implications of changes in aerobic capacity, strength and agility rather than evidence of a demonstrated reduction in injury occurrence.

## 4. Discussion

This systematic review examined the effects of high-intensity interval training (HIIT) on aerobic fitness, muscular strength and agility in football players. Overall, individual studies frequently reported favourable changes following HIIT-based interventions, but the strength of the evidence varied considerably across outcomes and should be interpreted cautiously.

For aerobic capacity, only two independent RCTs provided sufficiently comparable data for quantitative synthesis. Although the pooled estimate favoured HIIT (SMD = 0.51, 95% CI −0.03 to 1.05), the effect did not reach statistical significance (*p* = 0.064). The wide confidence interval and very wide prediction interval further indicate uncertainty regarding the magnitude and direction of effects that may occur in future studies. Therefore, the current evidence suggests a possible beneficial effect of HIIT on aerobic fitness but does not establish superiority over comparator training.

For muscular strength, favourable changes were reported in several individual outcomes, particularly where HIIT was combined with resistance training. However, an across-study meta-analysis was not possible because sufficiently comparable data from independent studies were unavailable. The four quantitative strength estimates reported by Zhang et al. [3] originated from a single-participant sample and were therefore interpreted as within-study effects rather than as a pooled treatment estimate. Consequently, the evidence regarding the independent effect of HIIT on muscular strength remains limited.

For agility, the pooled analysis of two RCTs favoured HIIT (SMD = 0.77, 95% CI 0.21 to 1.33; *p* = 0.0069). However, only two studies contributed to this estimate, and the very wide prediction interval indicates considerable uncertainty regarding the effect that might be observed in future populations. The agility findings should therefore be considered encouraging but preliminary rather than definitive.

These findings should also be considered in the context of the relatively small evidence base. Individual study sample sizes ranged from 16 to 54 participants, with a median sample size of approximately 34 participants. In addition, only two independent studies contributed to each eligible meta-analysis. These factors limit statistical power, precision and generalisability and are important when interpreting both statistically significant and non-significant findings.

### 4.1. Aerobic Capacity

Aerobic capacity demonstrated the most consistent and substantial improvements following high-intensity interval training (HIIT) interventions. Several studies [2,25] reported increases in VO_2_max of 12–17% after sprint-based or speed-based HIIT protocols, reflecting the robust central and peripheral adaptations elicited by this training method [11,28]. The extent of improvement appeared to depend on both the intervention duration and training frequency. Longer protocols, which typically last 8–10 weeks and are delivered two to three times per week, are associated with greater aerobic gains of up to 10–17% [8,14]. In contrast, shorter programmes of six weeks or less generally produced more modest improvements of approximately 5–7%, which is consistent with an early phase of adaptation that precedes more substantial long-term gains [1,3].

This pattern supports a dose–response relationship, where higher training frequency, longer exposure and greater accumulated high-intensity workload enhance cardiovascular adaptations such as increased cardiac output, capillary density and arterial compliance [28]. These physiological changes improve the ability of the cardiovascular system to deliver oxygen, leading to faster muscle and pulmonary VO_2_ kinetics and, consequently, higher VO_2_max values [28]. As a result, players can sustain high-intensity exercise for longer durations and recover more effectively between bouts of intense play [28].

Additional peripheral adaptations, including increased muscle oxidative and buffering capacities, may also occur through the upregulation of peroxisome proliferator-activated receptor gamma coactivator 1-alpha (PGC-1α), which is central to mitochondrial biogenesis and energy metabolism [2]. Together, these central and peripheral responses enhance oxygen delivery and utilisation, allowing players to maintain repeated high-intensity efforts and efficiently recover physiological capabilities that are critical for match performance [10,29].

However, the findings also highlight that training frequency and timing relative to the competitive season influence outcomes. Slettalokken et al. [26] reported maintenance rather than improvement in VO_2_max during the off-season, alongside a decline in shuttle run performance, likely reflecting a reduced overall training load and session frequency. This suggests that without adequate and sustained stimuli, aerobic adaptations may plateau or regress, underscoring the importance of maintaining conditioning throughout the annual training cycle.

Importantly, the quantitative evidence for aerobic capacity was less definitive than the individual study findings might suggest. Only two RCTs could be pooled, and although the pooled estimate favoured HIIT, the confidence interval crossed the null, and the effect was not statistically significant. The very wide prediction interval further indicates substantial uncertainty regarding the effect in future populations. Accordingly, the present review cannot establish that HIIT is superior to comparator training for improving aerobic capacity, and the apparent benefits observed in individual studies require confirmation in larger and methodologically robust trials.

### 4.2. Muscle Strength

Improvements in muscle strength were observed in all studies that assessed this outcome, with the most substantial gains reported when high-intensity interval training (HIIT) was combined with resistance training [24]. The magnitude of improvement ranged from moderate to large, depending on whether structured resistance training was incorporated. The largest increase, a 20.3% increase in one-repetition maximum (1-RM) half-square performance, was reported when concurrent HIIT was integrated into a preseason resistance training programme [24]. In contrast, shorter rehabilitation-focused HIIT interventions produced smaller strength gains of approximately 6–8%, likely reflecting limited mechanical loading and insufficient hypertrophic stimulus in both training designs [1,2,3].

According to concurrent training theory, performing large volumes of endurance or high-intensity work in close proximity to strength sessions without adequate recovery may lead to an interference effect that attenuates muscle hypertrophy and power development [7,8,9,10]. Studies that periodised HIIT and resistance training on alternate days or across separate microcycles reported superior neuromuscular adaptations compared with those that combined both modalities within a single session [1]. This suggests that structured periodisation, incorporating controlled sequencing, frequency and recovery, is essential for optimising both aerobic and strength adaptations in football players [7,24].

Collectively, the evidence indicates that integrating targeted resistance training with HIIT, while appropriately managing training load and recovery, can produce synergistic improvements in power output and muscular endurance. These adaptations are critical for explosive football-specific actions such as sprinting, jumping and tackling [10,24].

The improvement in muscle strength following HIIT appears to result primarily from neural adaptations, including enhanced motor unit recruitment and improved coordination between agonist and antagonist muscle groups [1]. Another plausible mechanism involves improved molecular signalling, such as increased mitochondrial biogenesis and increased substrate utilisation, including carbohydrate and fat oxidation [1]. High-intensity training does not necessarily increase the overall muscle cross-sectional area but selectively increases the proportion of type II fast-twitch fibres, which are responsible for rapid and powerful movements [3]. Furthermore, HIIT may enhance intermuscular coordination and neural drive, leading to more efficient force production during football-specific activities [3].

The strength evidence should also be interpreted cautiously. Although several favourable strength outcomes were reported, the multiple strength outcomes reported by Zhang et al. [3] originated from a single-participant sample and were therefore synthesised narratively rather than treated as independent quantitative effects. Consequently, no robust pooled estimate of the effect of HIIT on muscular strength can currently be established. Moreover, concurrent resistance training in some interventions makes it difficult to isolate the independent contribution of HIIT to observed strength gains.

### 4.3. Agility

Agility performance improved consistently across all included studies. The greatest enhancements were observed in sprint-based high-intensity interval training (HIIT) programmes that incorporated change-of-direction (COD) drills [25,27,30], which closely replicate the multidirectional and reactive movement demands of football. Studies have indicated that COD-integrated HIIT protocols combining 10–20 m sprints with multidirectional movements produced greater agility improvements than linear sprint formats did [27,28,31]. Gokkurt et al. [27] reported a small improvement in Pro-Agility Test performance, with completion time decreasing from 4.73 ± 0.39 s at baseline to 4.69 ± 0.39 s following the eight-week HIIT intervention, corresponding to an improvement of approximately 0.85%. Similarly, Wright et al. [28] reported agility enhancements of up to 7% in female footballers after a mixed-methods HIIT programme incorporating both linear and COD components. In contrast, a linear sprint HIIT model involving repeated 30 s runs at 90–95% HRmax improved agility by only 3–4%, reflecting limited transfer to COD performance [25].

These improvements are likely underpinned by neuromuscular adaptations such as enhanced coordination, improved proprioceptive feedback and faster motor unit recruitment, enabling athletes to execute rapid directional changes with greater efficiency and postural stability [26,27,28,30]. From an applied perspective, agility-focused HIIT may also enhance decision-making under physical stress, as players are required to perform directional changes in response to unpredictable stimuli that simulate real match situations [10,32].

The variability in outcomes across studies reinforces the principle of specificity and transfer to match demands, which posits that the closer the biomechanical and perceptual elements of training are to actual game conditions, the greater the gains in neuromuscular coordination and reactive agility [33,34]. Consequently, conditioning programmes that integrate sport-specific multidirectional drills are more effective for developing reactive agility and movement control than those relying solely on linear running intervals [27,28,34].

Although the pooled agility estimate was statistically significant, it was based on only two independent studies. The very wide prediction interval indicates substantial uncertainty regarding the magnitude and direction of effects that might be expected in future studies. Therefore, the finding should be regarded as preliminary rather than definitive, consistent with the low certainty assigned to this outcome using GRADE.

### 4.4. Injury-Related Implications and Evidence Gaps

None of the studies included in this systematic review directly evaluated injury incidence, injury burden, time-loss injury or reinjury as an outcome. Consequently, the present review provides no direct evidence that HIIT prevents injuries in football players, and changes in aerobic fitness, muscular strength or agility should not be interpreted as surrogate evidence of injury reduction.

The performance adaptations observed in the included studies may nevertheless have theoretical relevance to factors associated with injury mechanisms. For instance, improved aerobic fitness may contribute to greater fatigue tolerance, while increased muscular strength and improved movement control may influence an athlete’s capacity to tolerate football-specific physical demands [5,13,29,32,33,34,35,36,37]. These relationships, however, are derived from mechanistic reasoning and the wider injury-prevention literature rather than from direct injury outcomes in the studies included in this review.

Similarly, although concurrent HIIT and resistance training may improve physical characteristics relevant to football performance [24], the available evidence does not establish that these adaptations result in fewer injuries. The effects of HIIT on injury risk therefore remain uncertain.

Future trials should incorporate prospective injury surveillance using clearly defined outcomes such as injury incidence per exposure hours, injury burden, time-loss injury and reinjury, alongside measures of aerobic fitness, strength and neuromuscular performance. Such studies are required to determine whether changes in physical-performance characteristics following HIIT translate into clinically and practically meaningful reductions in football injury risk.

### 4.5. Strengths and Limitations

The strength of this review is the synthesis of evidence across multiple football-related performance domains, including aerobic fitness, muscular strength and agility, and the inclusion of both narrative and quantitative approaches where appropriate. The revised analysis also distinguishes between evidence derived from independent studies and multiple outcomes obtained within a single study, reducing the risk of overstating the strength of the quantitative evidence.

While this review provides strong preliminary evidence supporting the effectiveness of high-intensity interval training (HIIT) in football, several limitations within the current literature should be acknowledged. Most interventions last only 4–10 weeks, a duration sufficient to elicit early neural and metabolic adaptations but insufficient to capture long-term morphological changes such as muscle fibre hypertrophy, tendon stiffness or sustained neuromuscular control [3,24,38]. As football performance and injury resilience rely on these slower-developing traits, short-term HIIT studies may overestimate the immediate benefits while underrepresenting the durability of physiological gains [7,14]. The relatively small sample sizes of most included studies also limit their statistical power and precision, increasing the potential for random variation to influence observed outcomes [2]. Furthermore, representation of female football players remains limited. One study [2] reported a 12% improvement in VO_2_max among semi-professional female players following six weeks of heart rate- and speed-based HIIT, whereas another study [28] reported agility gains of 6–7% in adolescent girls after a mixed-method HIIT programme. These findings indicate differential responsiveness across performance domains and highlight the need for greater inclusion of women’s football, where hormonal fluctuations, sex-specific neuromuscular control and biomechanical factors such as joint laxity may influence both adaptation and injury risk [36,37].

Comparative analysis also suggested age- and maturity-related variability in responsiveness. Youth cohorts (U17–U19) demonstrated faster but smaller relative gains in VO_2_max (up to 8%) and agility (4–5%), whereas elite adult players required higher intensities to achieve modest improvements, typically 3–5% [1,12,14]. These contrasts imply that HIIT responsiveness is influenced by maturation and training status, underscoring the importance of individualising training load, intensity and recovery on the basis of developmental stage and competitive level [10,12].

An important limitation of the present review is the small evidence base available for quantitative synthesis. Only two independent studies contributed to each of the aerobic-capacity and agility meta-analyses, limiting the precision of pooled estimates and the reliability of heterogeneity statistics. Muscle-strength outcomes could not be meta-analysed across independent studies because the available quantitative estimates were derived primarily from multiple outcomes within a single study. Publication bias could not be meaningfully assessed because too few studies contributed to each synthesis. These limitations restrict the strength and generalizability of conclusions drawn from quantitative analyses.

Competitive level represents an additional limitation to the generalisability of the findings. Five of the ten included studies involved amateur or youth-amateur players, three involved semi-professional players, and only two involved professional players. Consequently, the available evidence is weighted towards developing and amateur football populations, and the extent to which the findings can be generalised to professional players remains uncertain. Although a subgroup analysis according to competitive level was considered, the small number of independent and outcome-comparable studies within each subgroup precluded a meaningful quantitative comparison. Training history, baseline fitness, accumulated training load and adaptation potential may differ substantially between amateur and professional players, and future adequately powered trials should therefore examine whether competitive level modifies the response to HIIT.

### 4.6. Practical Applications and Future Directions

On the basis of the available evidence, high-intensity interval training (HIIT) can be effectively incorporated into football conditioning programmes two to three times per week during the competitive season, with intensities maintained between 80% and 100% of the maximum heart rate (HRmax) to elicit both aerobic and anaerobic adaptations [2,7,14]. To maximise training efficiency, sessions should integrate aerobic endurance, muscular strength and agility components, which are structured according to positional demands and seasonal periodisation phases [10,24]. In rehabilitation settings, HIIT should be introduced progressively during the late recovery phase, as demonstrated in previous studies [1,3], ensuring that exercise complements sport-specific strength and neuromuscular control tasks. This approach facilitates gradual reconditioning while reducing the risk of reinjury [5].

From a coaching perspective, incorporating football-specific multidirectional drills and small-sided games within HIIT sessions can enhance transfer to match demands by improving reactive agility, movement control and decision-making under fatigue [27,28,34]. Monitoring player workload through heart rate, running velocity or ratings of perceived exertion is recommended to individualise training load and minimise the risk of overtraining [9].

HIIT represents a time-efficient and scalable conditioning strategy capable of improving cardiovascular fitness, muscular performance and movement efficiency while potentially reducing injury risk when integrated with balance and neuromuscular training [35]. To strengthen future research, HIIT interventions should adopt standardised reporting frameworks detailing work-to-rest ratios (1:1–1:3), frequencies (two to three sessions per week) and progression models to facilitate comparisons across studies [7,9]. Longer intervention periods (≥10–12 weeks) are also advised to capture sustained aerobic and neuromuscular adaptations that may not fully develop within shorter 4–8-week protocols [2,14].

Future trials should further examine sex-specific and age-specific responses, as the underrepresentation of female athletes currently limits the generalisability of findings to women’s football [2,14]. The inclusion of biomechanical and neuromuscular outcome measures alongside longitudinal injury surveillance would also clarify how HIIT influences load tolerance, coordination and fatigue resistance, thereby informing evidence-based approaches to injury prevention and performance enhancement in football [29,36].

## 5. Conclusions

This systematic review suggests that HIIT may improve several football-related physical-performance outcomes, including aerobic fitness, muscular strength and agility; however, the certainty and precision of the available evidence remain limited. Quantitative synthesis was constrained by the small number of independent studies, with no statistically significant pooled effect demonstrated for aerobic capacity and only two studies contributing to the significant pooled agility estimate. Muscle-strength findings could not be meta-analysed across independent studies.

Importantly, none of the included studies directly evaluated injury incidence or injury burden. Therefore, no conclusion can be drawn regarding the effectiveness of HIIT for injury prevention. Any potential relationship between HIIT-induced improvements in physical performance and injury-related mechanisms remains theoretical and requires direct investigation in appropriately designed prospective trials.

Key Points

- High-intensity interval training (HIIT) is an effective and time-efficient conditioning method for football players, producing meaningful improvements in aerobic capacity, muscular strength and agility across different age groups and competitive levels.
- HIIT programmes incorporating football-specific multidirectional drills and resistance components yield the greatest performance benefits and stronger transfer to match demands.
- Physiological adaptations associated with HIIT, including enhanced cardiovascular efficiency, neuromuscular control and fatigue resistance, may contribute to reduced injury risk during high-intensity play.
- To optimise outcomes, HIIT should be integrated two to three times per week within periodised football training frameworks, with appropriate load monitoring and progression to support both performance and rehabilitation goals.
- Further research is needed to standardise HIIT protocols, examine long-term adaptations and explore sex- and age-specific responses to improve evidence-based application in football conditioning and injury prevention.

## Figures and Tables

**Table 1:** Characteristics of the included studies.

No.	Author and Year	Study Type	Sample Size	Player Level	Age (Mean ± SD)	Type of Intervention	Tests Used	Outcomes Measured	Duration
1	Thomakos & Pierro’s (2024) [14]	RCT	26	Youth amateur	Exp: 18.7 ± 1.1 Ctrl: 18.1 ± 0.7	Control: Usual soccer training Experimental: 10 s/10 s linear running HIIT or 15 s/15 s COD HIIT at 100% MAS, twice per week	Yo-Yo Intermittent Recovery Test Level 1	VO_2_max	6 weeks
2	Zhang, Gaofei (2024) [3]	NRCT	50	Amateur	16.4 ± 1.3	Control: MICT (12 sessions) + resistance training Experimental: HIIT (12 sessions) + resistance training (squats, lunges, core exercises, crunches)	Isokinetic Strength Test	Body-mass-normalised isokinetic knee strength and power	4 weeks
3	Yan, Shuren (2022) [1]	NRCT	54	Amateur	Exp: 15.8 ± 0.9 Ctrl: 15.7 ± 0.8	Control: Low-frequency HIIT (2 sessions/week, 50–60 min) Experimental: High-frequency HIIT (5 sessions/week, 50–60 min)	Isokinetic Dynamometer	Body-mass-normalised isokinetic knee strength and power	4 weeks
4	Gokkurt, Kadir (2021) [27]	RCT	22	Semiprofessional	Exp: 17.55 ± 0.69 Ctrl: 18.36 ± 0.51	Control: Regular training Experimental: HIIT three times per week + regular training	Pro-Agility Test	Agility time (s)	8 weeks
5	Howard, Neal (2017) [25]	RCT	32	Amateur	Exp: 14.81 ± 1.22 Ctrl: 15.06 ± 0.77	Control: Team conditioning + endurance training (long runs, 3×/week) Experimental: HIIT (30 s sprints with 4.5 min recovery; 4 reps increasing to 6 reps by week 5)	1. Yo-Yo Intermittent recovery Test Level 1 2. Illinois Agility Test	1. VO_2_max 2. Agility (s)	10 weeks
6	Arazi, Hamid (2017) [2]	RCT	16	Semiprofessional	Exp: 23.4 ± 1.3 Ctrl: 23.4 ± 1.1	Control: HR-based HIIT (90% HRmax for 90 s, 90 s walk × 3 sequences/session) Experimental: Speed-based HIIT (90% max speed, 90 s work, 90 s walk × 2 sequences/session)	1. Hoff Test 2. 30–15 Intermittent Fitness Test	VO_2_max	6 weeks
7	Wright, Matthew (2016) [28]	RCT	37	Amateur	13.4 ± 1.5	Control: Football-specific training Experimental: HIIT	Modified T Test	Agility time (s)	8 weeks
8	Hamdani, Syed Ghayoor Ali (2014) [8]	NRCT	36	Professional	Exp: 26.1 ± 2.5 Ctrl: 25.4 ± 2.3	Control: Regular football training Experimental: HIIT	Beep Test	VO_2_max	8 weeks
9	Slettalokken, Gunnar (2014) [26]	RCT	17	Semiprofessional	Exp: 21.8 ± 2.2 Ctrl: 19.7 ± 3.1	HIT 0.5:1 HIIT session every second week; HIT 1:1 HIIT session per week. Both added to normal off-season activity	1. VO_2_max treadmill test 2. 20 m Shuttle Run	VO_2_max	6 weeks
10	Wong, Pui-Lam (2010) [24]	NRCT	39	Professional	Exp: 21.0 ± 1.0 Ctrl: 24.6 ± 1.5	Control: Regular soccer training Experimental: Strength training (2×/week; high pull, jump squat, bench press, back half squat, chin-ups, 4 sets × 6RM) + HIIT (15 s sprints × 16 intervals at 120% MAS with 15 s rest)	1RM Half Squat and Bench Press	Maximum weight lifted	8 weeks

Abbreviations: RCT, randomised controlled trial; NRCT, nonrandomized controlled trial; HIIT, high-intensity interval training; MICT, moderate-intensity continuous training; HRmax, maximum heart rate; MAS, maximal aerobic speed; COD, change in direction; VO_2_max, maximal oxygen uptake; 1RM, one repetition maximum; s, seconds; Exp, experimental; Ctrl, control.

**Table 2:** (**a**) Effects of high-intensity interval training on aerobic fitness and aerobic performance outcomes. (**b**) Effects of high-intensity interval training (HIIT) on muscle strength. (**c**) Effects of high-intensity interval training (HIIT) on agility performance.

(**a**)						
**Outcome Measure**	**Author**	**Test Used**	**Group**	**Pre (Mean ± SD)**	**Post (Mean ± SD)**	**Effect Size (Cohen’s d)**
Yo-Yo IR1 distance (m)	Thomakos & Pierro’s [14]	Yo-Yo Intermittent Recovery Test (YYIR1)	10 s/10 s HIIT	2083 ± 411	2434 ± 345	0.92
			15 s/15 s HIIT	2225 ± 262	2418 ± 275	0.72
Estimated VO_2_max (ml·kg^−1^·min^−1^)	Thomakos & Pierro’s [14]	Derived from Yo-Yo IR1	10 s/10 s HIIT	53.9 ± 3.5	56.8 ± 2.9	0.92
			15 s/15 s HIIT	55.1 ± 2.2	56.7 ± 2.3	0.72
VO_2_max (ml·kg^−1^·min^−1^)	Hamid Arazi [2]	Hoff Test	HR-based	48.6 ± 6.2	54.8 ± 10.6	0.59
			Speed-based	49.2 ± 4.1	53.6 ± 11.6	0.15
VO_2_max (mL·kg^−1^·min^−1^)	Syed Ghayoor Ali Hamdani [8]	VIFT/Beep Test	HR-based	50.6 ± 7.7	53.6 ± 8.2	0.59
			Speed-based	50.3 ± 6.1	59.0 ± 6.5	—
VO_2_max (mL·kg^−1^·min^−1^)	Gunnar Slettalokken [26]	Maximal treadmill VO_2_max test	HIT 0.5	63.4 ± 5.9	64.0 ± 5.9	—
			HIT 1	65.6 ± 2.1	64.3 ± 1.3	—
Yo-Yo IR1 distance (m)	Neal Howard [25]	Yo-Yo Intermittent Recovery Test (YYIR1)	Control	733.2 ± 318.8	1165.2 ± 252.8	—
			HIIT	741.6 ± 307.6	1067.6 ± 356.8	0.68
(**b**)						
**Outcome Measure**	**Author**	**Test Used**	**Group**	**Pre** **(Mean ± SD)**	**Post** **(Mean ± SD)**	**Effect Size (Cohen’s d)**
Isokinetic knee strength at 60°/s, body-mass-normalised	Gaofei Zhang [3]	Isokinetic Dynamometer—60°/s	HIIT	391.8 ± 42.1	431.9 ± 37.2	0.58
			MICT	388.8 ± 51.2	401.7 ± 44.4	—
Isokinetic knee power at 180°/s, body-mass-normalised	Gaofei Zhang [3]	Isokinetic Dynamometer—180°/s	HIIT	645.9 ± 72.4	695.1 ± 72.8	—
			MICT	636.6 ± 80.8	671.6 ± 82.8	—
Peak isokinetic knee torque at 60°/s, normalised	Shuren Yan [1]	Isokinetic Dynamometer	LFG	466.2 ± 58.9	482.7 ± 36.4	0.46
			HFG	464.9 ± 77.6	498.3 ± 64.5	—
Average isokinetic knee power at 180°/s, normalised	Shuren Yan [1]	Isokinetic Dynamometer	LFG	613.4 ± 95.5	632.9 ± 53.3	—
			HFG	629.8 ± 98.8	647.8 ± 90.3	—
1 Repetition Maximum (1RM)—Half Squat (kg)	Pui-Lam Wong [24]	1RM Strength Test	—	123.0 ± 1.5	148.0 ± 1.9	0.65–0.74
1 Repetition Maximum (1RM)—Bench Press (kg)	Pui-Lam Wong [24]	1RM Strength Test	—	65.3 ± 1.5	70.4 ± 1.1	0.65–0.74
(**c**)						
**Outcome Measure**	**Author**	**Test Used**	**Group**	**Pre** **(Mean ± SD)**	**Post** **(Mean ± SD)**	**Effect Size (Cohen’s d)**
Agility Time (s)	Kadir Gokkurt [27]	Pro-Agility Test	Experimental	4.73 ± 0.39	4.69 ± 0.39	0.88
			Control	4.56 ± 0.24	4.54 ± 0.24	—
Agility Time (s)	Neal Howard [25]	Illinois Agility Test	Experimental	16.26 ± 1.02	16.29 ± 0.92	0.66
			Control	16.67 ± 0.76	16.15 ± 0.49	—
Agility Time (s)	Matthew Wright [28]	Modified T Test	HIIT	7.12 ± 0.38	6.57 ± 0.35	0.57
			Control	—	—	—

Abbreviations: HIIT, high-intensity interval training; HR, heart rate; VO_2_max, maximal oxygen uptake; YYIR1, Yo-Yo Intermittent Recovery Test Level 1; VIFT, final velocity achieved in the 30–15 Intermittent Fitness Test where applicable; HIT 0.5, one high-intensity interval training session every second week; HIT 1, one high-intensity interval training session per week; m, metres; mL·kg^−1^·min^−1^, millilitres per kilogram per minute; MICT, moderate-intensity continuous training; LFG, low-frequency group; HFG, high-frequency group; Nm/kg, Newton metres per kilogram; 1RM, one repetition maximum; “—, not reported”; s, seconds. Note: The original studies reported body-mass-normalised isokinetic outcomes using inconsistent unit notation. Zhang et al. [3] described torque at 60°/s and power at 180°/s in the Methods but labelled both outcomes as Nm/kg in the results table. Yan et al. [1] reported normalised peak torque at 60°/s and average power at 180°/s using percentage-scaled values. To avoid unsupported unit conversion, values are reproduced as reported by the original studies and the measurement construct is specified separately.

**Table 3:** Quality assessment of included studies using the modified downs and black checklist.

Study	Study Design	Reporting (0–10)	External Validity (0–3)	Internal Validity—Bias (0–7)	Internal Validity—Confounding (0–6)	Power (0–2)	Total Score	Quality Rating
Thomakos & Pierro’s (2024) [14]	RCT	10	3	6	5	2	26	Excellent
Arazi, Hamid (2017) [2]	RCT	10	3	6	5	2	26	Excellent
Zhang, Gaofei (2024) [3]	NRCT	9	2	5	5	2	23	Good
Yan, Shuren (2022) [1]	NRCT	9	2	5	5	2	23	Good
Gokkurt, Kadir (2021) [27]	RCT	9	2	5	5	2	23	Good
Howard, Neal (2017) [25]	RCT	9	2	5	5	2	23	Good
Wright, Matthew (2016) [28]	RCT	9	2	5	5	2	23	Good
Slettalokken, Gunnar (2014) [26]	RCT	9	2	5	5	2	23	Good
Wong, Pui-Lam (2010) [24]	NRCT	8	2	4	3	2	19	Fair
Hamdani, Syed Ghayoor Ali (2014) [8]	NRCT	6	1	2	2	2	13	Poor

Abbreviations: RCT, randomised controlled trial; NRCT, non-randomised controlled trial.

**Table 4:** Summary of findings and certainty of evidence (GRADE).

Outcome	No. of Studies (Design)	Participants (n)	Effect Estimate/Synthesis Effect (Pooled SMD [95% CI])	Certainty of Evidence (GRADE)	Reasons for Downgrading
Aerobic capacity (VO_2_max)	5 studies: 4 RCTs [2,14,25,26] and 1 NRCT [8]	127	No pooled effect estimate. Individual studies reported favourable changes in aerobic fitness following HIIT, but effects varied according to intervention protocol, comparator condition and outcome measure. Some studies reported improvements in VO_2_max or Yo-Yo IR1 performance, whereas others demonstrated maintenance or no clear superiority over comparator training.	Low	Downgraded for methodological limitations and imprecision. Evidence was derived from small studies with heterogeneous HIIT protocols, comparator conditions and aerobic-fitness measures. One study was non-randomised and rated poor methodological quality. Outcome measures included directly or indirectly estimated VO_2_max and field-based aerobic-performance tests, limiting comparability. Source-verifiable data were insufficiently comparable for reliable quantitative pooling, and superiority of HIIT over comparator training was not consistently demonstrated.
Muscle strength (isokinetic torque/1-RM)	3 NRCTs [1,3,24]	143 participants across studies	No pooled estimate. Narrative evidence reported favourable changes in selected strength outcomes, but findings could not be quantitatively pooled because of heterogeneous assessment methods and intervention characteristics.	Low	Evidence derived entirely from non-randomised studies. Strength outcomes were measured using different methods, including isokinetic dynamometry and 1RM testing. Some interventions combined HIIT with resistance training, limiting attribution of effects specifically to HIIT. Small study samples and the absence of independent comparable datasets are further reduced certainty.
Agility performance (time, s)	2 RCTs [25,27,28] narrative only	54 in quantitative synthesis	−0.29 [−0.83 to 0.25]	Low	Downgraded for imprecision because only two small RCTs contributed to the pooled estimate and the CI crossed the null. The corrected analysis did not demonstrate superiority of HIIT over comparator training.
Injury prevention (indirect evidence)	No direct eligible outcome data	Not applicable	Not applicable	Not graded	No included study directly quantified injury incidence. Injury-prevention implications are therefore indirect.

Abbreviations: SMD, standardised mean difference; CI, confidence interval; VO2max, maximal oxygen uptake; RCT, randomised controlled trial; NRCT, non-randomised controlled trial; HIIT, high-intensity interval training; Yo-Yo IR1, Yo-Yo Intermittent Recovery Test Level 1; 1RM, one-repetition maximum.

## Data Availability

The data supporting the findings of this systematic review and meta-analysis were obtained from the published studies cited in the manuscript. The extracted data used in the analyses are available within the article and may also be obtained from the corresponding author upon reasonable request.

## Abbreviations

The following abbreviations are used in this manuscript:

1RM	One-repetition maximum
ACL	Anterior cruciate ligament
CI	Confidence interval
COD	Change of direction
ES	Effect size
GRADE	Grading of Recommendations Assessment, Development and Evaluation
HFG	High-frequency group
HIIT	High-intensity interval training
HIT	High-intensity training
HR	Heart rate
HRmax	Maximum heart rate
I^2^	I-squared statistic
JBI	Joanna Briggs Institute
LFG	Low-frequency group
MAS	Maximal aerobic speed
MICT	Moderate-intensity continuous training
NRCT	Non-randomised controlled trial
OSF	Open Science Framework
PGC-1α	Peroxisome proliferator-activated receptor gamma coactivator 1-alpha
PRISMA	Preferred Reporting Items for Systematic Reviews and Meta-Analyses
RCT	Randomised controlled trial
RoB 2	Risk of Bias 2
ROBINS-I	Risk of Bias in Non-randomised Studies of Interventions
SMD	Standardised mean difference
VIFT	Vameval Intermittent Fitness Test
VO_2_max	Maximal oxygen uptake

## Appendix A

**Table A1:** PRISMA 2020 Checklist.

Section and Topic	Item #	Checklist Item	Location Where Item Is Reported
**TITLE**			
Title	1	Identify the report as a systematic review.	Pg No. 1
**ABSTRACT**			
Abstract	2	See the PRISMA 2020 for Abstracts checklist.	Pg No. 2
**INTRODUCTION**			
Rationale	3	Describe the rationale for the review in the context of existing knowledge.	Pg Nos. 3,4
Objectives	4	Provide an explicit statement of the objective(s) or question(s) the review addresses.	Pg No. 4
**METHODS**			
Eligibility criteria	5	Specify the inclusion and exclusion criteria for the review and how studies were grouped for the syntheses.	Pg Nos. 4,5
Information sources	6	Specify all databases, registers, websites, organisations, reference lists and other sources searched or consulted to identify studies. Specify the date when each source was last searched or consulted.	Pg No. 4
Search strategy	7	Present the full search strategies for all databases, registers and websites, including any filters and limits used.	Pg No. 4
Selection process	8	Specify the methods used to decide whether a study met the inclusion criteria of the review, including how many reviewers screened each record and each report retrieved, whether they worked independently, and if applicable, details of automation tools used in the process.	Pg No. 4
Data collection process	9	Specify the methods used to collect data from reports, including how many reviewers collected data from each report, whether they worked independently, any processes for obtaining or confirming data from study investigators, and if applicable, details of automation tools used in the process.	Pg Nos. 5,7
Data items	10a	List and define all outcomes for which data were sought. Specify whether all results that were compatible with each outcome domain in each study were sought (e.g., for all measures, time points, analyses), and if not, the methods used to decide which results to collect.	Pg Nos. 5,7,8
	10b	List and define all other variables for which data were sought (e.g., participant and intervention characteristics, funding sources). Describe any assumptions made about any missing or unclear information.	Pg No. 5
Study risk of bias assessment	11	Specify the methods used to assess risk of bias in the included studies, including details of the tool(s) used, how many reviewers assessed each study and whether they worked independently, and if applicable, details of automation tools used in the process.	Pg Nos. 5,6,7
Effect measures	12	Specify for each outcome the effect measure(s) (e.g., risk ratio, mean difference) used in the synthesis or presentation of results.	Pg Nos. 7,8
Synthesis methods	13a	Describe the processes used to decide which studies were eligible for each synthesis (e.g., tabulating the study intervention characteristics and comparing against the planned groups for each synthesis (item #5)).	Pg Nos. 8,9,10,11
	13b	Describe any methods required to prepare the data for presentation or synthesis, such as handling of missing summary statistics, or data conversions.	Pg Nos. 8,9
	13c	Describe any methods used to tabulate or visually display results of individual studies and syntheses.	Pg Nos. 10,11
	13d	Describe any methods used to synthesise results and provide a rationale for the choice(s). If meta-analysis was performed, describe the model(s), method(s) to identify the presence and extent of statistical heterogeneity, and software package(s) used.	Pg No. 15
	13e	Describe any methods used to explore possible causes of heterogeneity among study results (e.g., subgroup analysis, meta-regression).	Pg No. 15
	13f	Describe any sensitivity analyses conducted to assess robustness of the synthesised results.	NA
Reporting bias assessment	14	Describe any methods used to assess risk of bias due to missing results in a synthesis (arising from reporting biases).	Pg Nos. 5,6,7
Certainty assessment	15	Describe any methods used to assess certainty (or confidence) in the body of evidence for an outcome.	NA
**RESULTS**			
Study selection	16a	Describe the results of the search and selection process, from the number of records identified in the search to the number of studies included in the review, ideally using a flow diagram.	Pg Nos. 8,9
	16b	Cite studies that might appear to meet the inclusion criteria, but which were excluded, and explain why they were excluded.	Pg No. 9
Study characteristics	17	Cite each included study and present its characteristics.	Pg Nos. 10,11
Risk of bias in studies	18	Present assessments of risk of bias for each included study.	Pg Nos.5,6,7
Results of individual studies	19	For all outcomes, present, for each study: (a) summary statistics for each group (where appropriate) and (b) an effect estimate and its precision (e.g., confidence/credible interval), ideally using structured tables or plots.	Pg Nos. 13,14,16–20
Results of syntheses	20a	For each synthesis, briefly summarise the characteristics and risk of bias among contributing studies.	Pg Nos. 16–20
	20b	Present results of all statistical syntheses conducted. If meta-analysis was done, present for each the summary estimate and its precision (e.g., confidence/credible interval) and measures of statistical heterogeneity. If comparing groups, describe the direction of the effect.	Pg Nos. 16–20
	20c	Present results of all investigations of possible causes of heterogeneity among study results.	Pg Nos. 16–20
	20d	Present results of all sensitivity analyses conducted to assess the robustness of the synthesised results.	NA
Reporting biases	21	Present assessments of risk of bias due to missing results (arising from reporting biases) for each synthesis assessed.	Pg Nos. 5,6,7
Certainty of evidence	22	Present assessments of certainty (or confidence) in the body of evidence for each outcome assessed.	NA
**DISCUSSION**			
Discussion	23a	Provide a general interpretation of the results in the context of other evidence.	Pg Nos. 20–23
	23b	Discuss any limitations of the evidence included in the review.	Pg Nos. 23,24
	23c	Discuss any limitations of the review processes used.	Pg Nos. 23,24
	23d	Discuss implications of the results for practice, policy, and future research.	Pg Nos. 24,25
**OTHER INFORMATION**			
Registration and protocol	24a	Provide registration information for the review, including register name and registration number, or state that the review was not registered.	Pg No. 4
	24b	Indicate where the review protocol can be accessed, or state that a protocol was not prepared.	NA
	24c	Describe and explain any amendments to information provided at registration or in the protocol.	NA
Support	25	Describe sources of financial or nonfinancial support for the review, and the role of the funders or sponsors in the review.	Pg No. 26
Competing interests	26	Declare any competing interests of review authors.	Pg No. 26
Availability of data, code and other materials	27	Report which of the following are publicly available and where they can be found: template data collection forms; data extracted from included studies; data used for all analyses; analytic code; any other materials used in the review.	NA

From: Page, M.J.; McKenzie, J.E.; Bossuyt, P.M.; Boutron, I.; Hoffmann, T.C.; Mulrow, C.D.; Shamseer, L.; Tetzlaff, J.M.; Akl, E.A.; Brennan, S.E.; et al. The PRISMA 2020 statement: an updated guideline for reporting systematic reviews. *BMJ* **2021**, *372*, n71. https://doi.org/10.1136/bmj.n71. This work is licenced under CC BY 4.0. To view a copy of this licence, visit https://creativecommons.org/licences/by/4.0/, accessed on 27 August 2026 [19].

**Table A2:** Complete Database-Specific Search Strategies.

Source	Search Strategy	Limits
**PubMed/MEDLINE**	(“high-intensity interval training”[Title/Abstract] OR “high intensity interval training”[Title/Abstract] OR HIIT[Title/Abstract] OR “interval training”[Title/Abstract]) AND (football[Title/Abstract] OR footballer*[Title/Abstract] OR soccer[Title/Abstract] OR “soccer player”[Title/Abstract] OR “soccer players”[Title/Abstract]) AND (“aerobic capacity”[Title/Abstract] OR “aerobic fitness”[Title/Abstract] OR VO2max[Title/Abstract] OR “maximal oxygen uptake”[Title/Abstract] OR “muscle strength”[Title/Abstract] OR “muscular strength”[Title/Abstract] OR “isokinetic strength”[Title/Abstract] OR agility[Title/Abstract] OR “change of direction”[Title/Abstract])	1 January 2010–31 December 2024
**CINAHL Plus, EBSCOhost**	S1: TI/AB (“high-intensity interval training” OR “high intensity interval training” OR HIIT OR “interval training”); S2: TI/AB (football OR footballer* OR soccer OR “soccer player*”); S3: TI/AB (“aerobic capacity” OR “aerobic fitness” OR VO2max OR “maximal oxygen uptake” OR “muscle strength” OR “muscular strength” OR “isokinetic strength” OR agility OR “change of direction”); S4 = S1 AND S2 AND S3	January 2010–December 2024
**SPORT Discus, EBSCOhost**	S1: TI/AB (“high-intensity interval training” OR “high intensity interval training” OR HIIT OR “interval training”); S2: TI/AB (football OR footballer* OR soccer OR “soccer player*”); S3: TI/AB (“aerobic capacity” OR “aerobic fitness” OR VO2max OR “maximal oxygen uptake” OR “muscle strength” OR “muscular strength” OR “isokinetic strength” OR agility OR “change of direction”); S4 = S1 AND S2 AND S3	January 2010–December 2024
**ProQuest**	(“high-intensity interval training” OR “high intensity interval training” OR HIIT OR “interval training”) AND (football OR footballer* OR soccer OR “soccer player*”) AND (“aerobic capacity” OR “aerobic fitness” OR VO2max OR “maximal oxygen uptake” OR “muscle strength” OR “muscular strength” OR “isokinetic strength” OR agility OR “change of direction”)	January 2010–December 2024
**Sports Medicine and Education Index, ProQuest**	Same three-concept Boolean strategy as ProQuest above	January 2010–December 2024
**Google Scholar**	Three searches conducted separately for aerobic fitness, muscle strength and agility using the HIIT + football/soccer concept with the corresponding outcome terms shown above	Custom range 2010–2024

**Table A3:** JBI Data Extraction Form for Systematic Reviews and Research Synthesis.

Study Details	
Author/year	
objectives	
Participants (characteristics/ total number)	
Setting/context	
Description of Interventions/ phenomena of interest	
Search Details	
Sources searched	
Range (years) of included studies	
Number of studies included /	
Types of studies included	
Country of origin of included studies	
Appraisal	
Appraisal instruments used	
Appraisal rating	
Analysis	
Method of analysis	
Outcome assessed	
Results/Findings	
Significance/direction	
Heterogeneity	
Comments	
